# Mechanochemical Synthesis and Physicochemical Characterization of Previously Unreported Praziquantel Solvates with 2-Pyrrolidone and Acetic Acid

**DOI:** 10.3390/pharmaceutics13101606

**Published:** 2021-10-02

**Authors:** Debora Zanolla, Lara Gigli, Dritan Hasa, Michele R. Chierotti, Mihails Arhangelskis, Nicola Demitri, William Jones, Dario Voinovich, Beatrice Perissutti

**Affiliations:** 1 Department of Chemical and Pharmaceutical Sciences, University of Trieste, Piazzale Europa 1, 34127 Trieste, Italy; debora.zanolla@gmail.com (D.Z.); dhasa@units.it (D.H.); 2 Elettra-Sincrotrone Trieste, S.S. 14 Km 163.5, Area Science Park, Basovizza, 34149 Trieste, Italy; lara.gigli@elettra.eu (L.G.); nicola.demitri@elettra.eu (N.D.); 3 Department of Chemistry and NIS Centre, University of Torino, V. Giuria 7, 10125 Torino, Italy; michele.chierotti@unito.it; 4 Faculty of Chemistry, University of Warsaw 1 Pasteura Street, 02-093 Warsaw, Poland; m.arhangelskis@uw.edu.pl; 5 Department of Chemistry, University of Cambridge, Lensfield Road, Cambridge CB2 1EW, UK; wj10@cam.ac.uk

**Keywords:** praziquantel, solvates, acetic acid, 2-pyrrolidone, mechanochemistry, liquid-assisted grinding

## Abstract

Two new solvates of the widely used anthelminthic Praziquantel (PZQ) were obtained through mechanochemical screening with different liquid additives. Specifically, 2-pyrrolidone and acetic acid gave solvates with 1:1 stoichiometry (PZQ-AA and PZQ-2P, respectively). A wide-ranging characterization of the new solid forms was carried out by means of powder X-ray diffraction, differential scanning calorimetry, FT-IR, solid-state NMR and biopharmaceutical analyses (solubility and intrinsic dissolution studies). Besides, the crystal structures of the two new solvates were solved from their Synchrotron-PXRD pattern: the solvates are isostructural, with equivalent triclinic packing. In both structures acetic acid and 2-pyrrolidone showed a strong interaction with the PZQ molecule via hydrogen bond. Even though previous studies have shown that PZQ is conformationally flexible, the same *syn* conformation as the PZQ Form A of the C=O groups of the piperazinone-cyclohexylcarbonyl segment is involved in these two new solid forms. In terms of biopharmaceutical properties, PZQ-AA and PZQ-2P exhibited water solubility and intrinsic dissolution rate much greater than those of anhydrous Form A.

## 1. Introduction

**Scheme 1:**
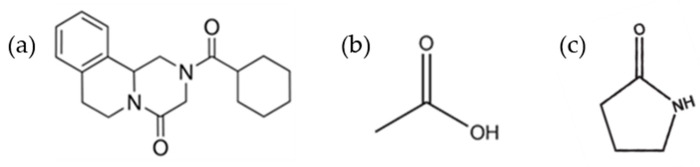
Chemical structure of Praziquantel (**a**), acetic acid (**b**) and 2-pyrrolidone (**c**).

**Figure 1:**
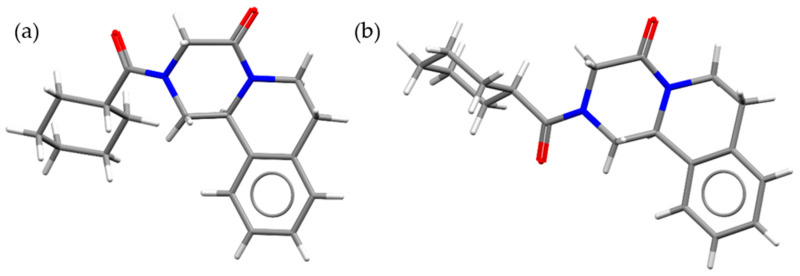
Molecule of PZQ in (**a**) *syn* and (**b**) *anti* conformations.

Praziquantel (PZQ, Scheme 1), ((11bRS)-2-(Cyclohexylcarbonyl)-1,2,3,6,7,11b-hexahydro-4-Hpyrazino [2,1-a]isoquinolin-4-one), is the first-line drug to treat human schistosomiasis [1,2], a tropical disease caused by the Schistosoma species worm, which affects around 240 million people worldwide and more than 700 million people live in endemic areas [3]. For this reason, PZQ is included in World Health Organization (WHO) Model List of Essential Medicines for Adults (21st list available at [4]) and for Children (7th list available at [5]). The commercial form of PZQ is a racemic anhydrate structure, indexed as TELCEU in the Cambridge Structural Database (CSD) [6]. Pure enantiomers can be also crystallized separately as hemihydrate forms (CSD codes SIGBUG, SIGBUG01) [7,8]. PZQ possesses two additional anhydrate polymorphic forms (Form B and Form C, indexed as TELCEU01 and GOYZOM, respectively), obtainable via neat grinding PZQ Form A [9,10]. Despite the absence of hydrogen bond donor groups in the molecular structure (Scheme 1), PZQ shows an interesting propensity to form a variety of multicomponent systems. A series of PZQ cocrystals with different dicarboxylic acids (i.e., oxalic, malonic, succinic, maleic, fumaric, glutaric, adipic and pimelic) were attained by means of liquid-assisted grinding (LAG) with acetone or acetonitrile. Recently, our group reported a hemihydrate form of racemic PZQ (indexed as WUHQAU), obtained via LAG in a two-step mechanochemical treatment when the commercial PZQ (Form A) is used, or in a one-step in the case of PZQ Form B [11]. Both in the case of PZQ cocrystals with dicarboxylic acids and in the racemic hemihydrate, the PZQ adopts an *anti*-conformation of the C=O groups of the piperazinone-cyclohexylcarbonyl segment, whereas commercially available PZQ Form A has the *syn* stereochemistry [6,11] (Figure 1).

New solvates are discovered either by chance during a specific manufacturing process or through systematic polymorph screening programs. Most screening methods are based on solution crystallization techniques (i.e., in the presence of excess solvent) where only the most stable solvate is likely to be obtained [12]. In this study, we explore the use of mechanochemistry, which emerged as particularly prominent method [13] for the discovery of multicomponent molecular solids [14]. In this context, one can find grinding in the presence of a minor quantity of a certain solvent [15]. This technique, as might be expected, seems to provide greater molecular mobility than neat grinding, thus it is promising for the discovery of alternative crystalline forms. This method has also been documented as effective for inducing specific polymorph transformations and for discovering different solid forms (including new crystal forms—e.g., cocrystals—that cannot be obtained easily by alternative approaches) [16]. Even though a few systematic studies about the mechanochemical synthesis of solvates are reported [17,18,19,20], the solvate outcome is often regarded as an undesired by-product rather than its main goal [16,21].

Here we present a mechanochemical screening study for the search of possible solvated forms of PZQ, which, as previously said, has displayed an interesting solid-state behavior, forming a variety of anhydrous and hydrate forms by mechanochemistry, and is thus an excellent candidate for the formation of novel multicomponent crystalline phases. The knowledge on its crystalline features and the discovery of new crystalline phases might also help overcoming some of the limitations related to this important and essential drug for the health of humans and animals. Further, in the case of PZQ, the knowledge about the existence of solvated forms is paramount, due to the fact that, in pharmaceutical industry, solution crystallization is a critical step for the manufacturing and production of PZQ, and PZQ tablets are commonly manufactured also using wet granulation process, including in some cases the use of organic solvent-based wet granulation [22].

We survey the solid form landscape of PZQ by mechanochemistry in the presence of eight commonly used solvents namely nitromethane, acetonitrile, 1,4-dioxane, ethyl acetate, ethanol, 2-pyrrolidone, acetic acid and methanol. Of these, only acetic acid and 2-pyrrolidone (whose structures are also reported in Scheme 1) gave two new solid forms, both monosolvates (PZQ-AA and PZQ-2P). The two solvates were fully characterized, revealing some interesting solid-state features and in vitro dissolution profiles.

## 2. Materials and Methods

### 2.1. Materials

Praziquantel (PZQ, (11bRS)-2-(Cyclohexylcarbonyl)-1,2,3,6,7,11b-hexahydro-4-H-pyrazino[2,1-a]isoquinolin-4-one)) was of Ph. Eur. grade and kindly donated by Fatro S.p.a. (Bologna, Italy). Nitromethane, acetonitrile, 1,4-dioxane, ethyl acetate, ethanol, methanol, 2-pyrrolidone and acetic acid of reagent grade were from SIGMA-Aldrich (Milano, Italy). The HiPersolv Chromanorm methanol used for the HPLC analysis was purchased from VWR Chemicals (BHD PROLABO, Milano, Italy).

### 2.2. Preparation of Praziquantel Solvates

The grinding experiments were performed using a Retsch vibrational mill MM200 (Retsch, Germany) and stainless steel jars of 25 mL with two stainless steel balls with a diameter of 7 mm. PZQ Form A, commercially available, was accurately weighted (200 mg) and shaken for 30 min at 25 Hz in the presence of a suitable liquid (ranging between 10 and 100 µL, corresponding to a η from 0.05 to 0.5 µL mg^−1^) [23]. Snap closed grinding jars were used to minimize liquid evaporation. After a set of preliminary trials with several solvents (nitromethane, acetonitrile, 1,4-dioxane, ethyl acetate, methanol, ethanol, 2-pyrrolidone and acetic acid) 2-pyrrolidone and acetic acid were used for the preparation of PZQ-2P and PZQ-AA, respectively, starting from the commercial form of PZQ (Form A). Each experiment was carried out at least twice to ensure reproducibility. In a typical 30 min LAG experiment at 25 Hz, from a starting temperature ranging about 25 °C, the increase in the temperature of the reaction mixture during grinding was typically around 5 °C. Powder temperature inside the jar was measured as previously reported [24].

The same PZQ-2P and PZQ-AA could be also obtained through a LAG process in the presence of 2-pyrrolidone and acetic acid starting with PZQ Form B. Anhydrous Form B was previously synthetized by neat grinding as reported in foregoing studies [9]. Besides, unsuccessful LAG experiments were conducted in the presence of ethanol and ethyl acetate from Form B. Similarly to the case of PZQ hemihydrate [11], the quantity of liquid added was not significant, since the same patterns were collected in all the experiments. Therefore, to simplify the preparation of the sample and the relative comparison, an equimolar ratio between the components was selected as the main production method in the case of Form B.

For comparison purposes, slurry experiments were also performed on Form A in the presence of acetic acid and 2-pyrrolidone. Each experiment was carried out at least twice. A large excess of solid was added to 3 mL of each solvent and left under stirring at room temperature in screw-cap vials for 7 days and the solid samples were checked by powder X-ray diffraction. Specifically, the PZQ concentration of the suspensions was 0.77 g/mL and 0.17 g/mL in the acetic acid and 2-pyrrolidone, respectively.

### 2.3. Characterization of Praziquantel Solvates

#### 2.3.1. Thermal Analyses

Differential Scanning Calorimetry (DSC) was conducted using a Mettler Toledo DSC822e system; about 2 mg of the samples were accurately weighted, put in a 40 μL aluminum perforated crucible and analyzed by heating from 30 to 160 °C at 10 °C/min under nitrogen atmosphere. For the hot stage microscopy (HSM) analysis, some grains of powder were positioned in a microscope slide and then observed upon heating from RT to about 150 °C in a hot stage system Mettler Toledo FP5. PZQ Form A, PZQ-AA and PZQ-2P were observed using a Reichert Biovar microscope with micro ocular MD-300 and using the software Webcam Companion for data collection.

#### 2.3.2. FT-IR Spectroscopy

The samples were analyzed using a Perkin-Elmer System 2000 FT-IR instrument on compressed disc prepared by gently grinding the sample in an agate mortar with anhydrous KBr (in a sample to KBr wt ratio of 1:15) and tableting with a hydraulic press (PerkinElmer, Norwalk, CA, USA). The range observed was from 400 to 4000 cm^−1^, the resolution used was 4 cm^−1^ with a step of 1 cm^−1^ and scan number of 3.

#### 2.3.3. Powder X-ray Diffraction (PXRD)

Powder X-Ray Diffraction analyses were conducted by means of a Panalytical X’Pert Pro Diffractometer, with a RTMS X’celerator detector (Panalytical, Almelo, Netherlands) and a Ni-filtered Cu Kα radiation (λ = 1.5418 Å). About 20 mg of the sample was gently pressed on a sample holder with a glass slide to give a flat surface and subsequently analyzed. The data were collected in the 2θ range of 3–40° using a step size of 0.0334° and a scan speed of 0.142° s^−1^.

#### 2.3.4. Synchrotron X-ray Powder Diffraction and Solvate Structure Solution

The diffraction patterns of PZQ-AA and PZQ-2P were collected at the X-ray diffraction beamline (XRD1) of Elettra Synchrotron, Trieste, Italy [25,26] in transmission mode, at room temperature (25 °C) with a monochromatic wavelength of 0.700 Å (17.71 KeV) using a Pilatus 2M hybrid-pixel area detector (Dectris, Baden-Daettwil, Switzerland) at a working distance of 400 mm, using a 100 µm aperture. Both the solvated forms have been packed in borosilicate capillaries with a 500 µm diameter (10 µm wall thickness). The two-dimensional powder patterns have been integrated using Fit2D program (2018, ESRF, Grenoble, France) [26,27], after preliminary calibration of hardware setup, using a capillary (Hilgenberg GmbH, Malsfeld, Germany) filled with LaB_6_ standard reference powder (NIST 660a, National institute of Standard and Thecnology, U.S. Department of Commerce).

The structure of the two solvated forms were solved by direct space methods, through a simulated annealing (SA) protocol using EXPO2014 [28]. The starting structural model containing one TELCAQ molecule [29] and one molecule of acetic acid and one of 2-pyrrolidone was built with Material studio software v 7.0. (MS) (Biovia, San Diego, USA) [30] and then geometrically optimized by the Dmol3 code implemented in MS. During the SA the cyclohexyl group of the PZQ molecule was allowed to freely rotate.

#### 2.3.5. Scanning Electron Microscopy (SEM)

A JEOL JSM-5510LV Scanning Electron Microscope (Jeol Ltd, Welwyn, UK) was used for imaging on gold metallized samples.

#### 2.3.6. Solid-State NMR Measurements

Solid-state NMR (SSNMR) measurements were collected on a Bruker Advance II 400 instrument (Bruker GmbH, Manheim, Germany) operating at 400.23 and 100.65 MHz for ^1^H and ^13^C nuclei, respectively. Cylindrical zirconia rotors (4 mm o.d.) were used with a sample volume of 80 µL and spun at 12 kHz. In all the analyses a RAMP-CP pulse sequence (^1^H 90° pulse = 3.05 μs), TPPM ^1^H decoupling with a radio frequency field of 75 kHz were used. In total, 124 transients were acquired with 3 ms of contact time and a relaxation delays of 20 s. The chemical shift scale was referenced through the resonance of glycine (^13^C methylene signal at 43.5 ppm) which was used as an external standard.

#### 2.3.7. Periodic DFT Geometry Optimization

Periodic density-functional theory (DFT) calculations were used to verify the accuracy of the crystal structure determinations from powder X-ray data, as well as to simulate the solid-state NMR spectra. The calculations were performed using a plane-wave DFT code CASTEP19 [31]. The experimental crystal structures of PZQ-AA and PZQ-2P were converted into CASTEP input format using the program cif2cell [32].

Geometry optimization of the crystal structures was carried out with PBE [33] functional combined with many-body dispersion (MBD*) [34,35,36] semiempirical correction scheme. The plane wave basis set was truncated at 650 eV cutoff and ultrasoft on-the-fly generated pseudopotentials were used. The 1st electronic Brillouin zone was sampled with a 2π × 0.05 Å^−1^ k-point spacing. The geometry optimization was performed in a 2-step process: first the atom coordinates were optimized while keeping the unit cell parameters fixed at their experimental values; in the subsequent optimization step the unit cell parameters were optimized together with the atom coordinates. Throughout both optimization steps the crystal structures were subjected to the space group symmetry constraints. Geometry optimization was conducted under the following convergence criteria: maximum total energy change 2 × 10^−5^ eV/atom; maximum atom displacement 10^−3^ Å; maximum atomic force 0.05 eV Å^−1^; residual stress 0.05 GPa. The residual stress criterion was only used in the case of variable cell geometry optimization.

The geometry-optimized structures were compared with their experimental counterparts, and the root mean square Cartesian displacement (RMSCD) was found to be 0.294 for PZQ-AA and 0.24 for PZQ-2P, both within the 0.35 Å limit signifying reliable structure determination [37].

#### 2.3.8. GIPAW-DFT

The optimized crystal structures were used for modelling SSNMR spectra. NMR parameters were calculated using the CASTEP implementation of GIPAW method [38]. The plane wave cutoff was set to 1000 eV, the standard and fine FFT grid scales were set to 2 and 3, respectively. All other calculation parameters remained the same as for the geometry optimization. The calculated chemical shieldings were converted into chemical shift using a reference shielding of 170 ppm. The spectral lines were drawn with Lorentzian curves with 1 ppm HWHM.

#### 2.3.9. Water Solubility and Intrinsic Dissolution Rate

The solubility of the samples was analyzed by preparing 10 mL of saturated solutions of each sample in distilled water, kept under agitation in the dark for 48 h. Then, the solutions were filtered using a membrane (pore size 0.45 µm) and diluted 1:200 with the mobile phase prior to injection, using the previously reported HPLC method [9]. Each analysis was conducted in triplicate and the average was reported.

For the intrinsic dissolution rate determinations, about 150 mg of the samples were inserted in the sample holder and pressed using a hydraulic press (PerkinElmer, Norwalk, CA, USA) for 1 min at 1 ton. The sample surface area obtained was of 0.785 cm^2^ and the entire sample holder with the compressed powder was dipped in a vessel containing 1 L of distilled water kept at 37 °C. The system used was a Hanson Research SR8 Plus dissolution test station and the paddles were positioned at 3.5 cm from the tablet surface, with a rotation speed of 100 rpm. About 2 mL of the dissolution medium were withdrawn every ten minutes till 60 min and immediately replaced with an equal amount of thermostated distilled water. After a 1:20 dilution with the mobile phase, the aliquots were analyzed using the same HPLC method as the solubility measurements. Triplicated analyses were performed and the mean (±S.D.) was calculated at each time point. The amount of the dissolved drug per unit area over time was indicated by the slope of the curves, obtained through a linear regression method. The dissolution behavior of the samples was compared and the curves were considered statistically different when *p* values did not exceed 0.05.

#### 2.3.10. Physical Stability

PZQ-AA and PZQ-2P (2 batches each) were kept in a desiccator at room temperature in the dark. Every month over a period of 18 months, solid samples were retrieved and analyzed via PXRD measurements.

Further, PZQ-AA and PZQ-2P physical stability upon thermal and mechanical treatment was tested. In particular, the monosolvated samples were heated at constant temperature of 50 °C under vacuum over-night and the obtained product assayed by PXRD. PZQ-AA and PZQ-2P were also ground for 200 min at 25 Hz and again their solid state was assessed by means of PXRD.

## 3. Results

A crystal form screening of PZQ was conducted by milling pure anhydrous PZQ Form A in the presence of eight commonly used solvents. The solvents, their main physicochemical parameters and the outcomes are reported in Table 1. It can be seen, besides the pure anhydrous PZQ Form A, that two new crystalline forms are obtained from acetic acid and 2-pyrrolidone. PXRD measurements, SEM analyses and thermal methods were applied to characterize the obtained crystal forms. It was found that all the new crystal forms should be assigned to PZQ solvates, which were labeled as PZQ-AA and PZQ-2P, respectively. These liquid additives enabled the formation of the same solvates when PZQ Form B was used as starting material (as also reported in Table 1). Moreover, the formation of the new phases was complete, since neither with PZQ A nor with PZQ B evidence of the starting materials was found. Slurry experiments of PZQ Form A suspended in acetic acid and 2-pyrrolidone gave origin to the same solvates.

**Figure 2:**
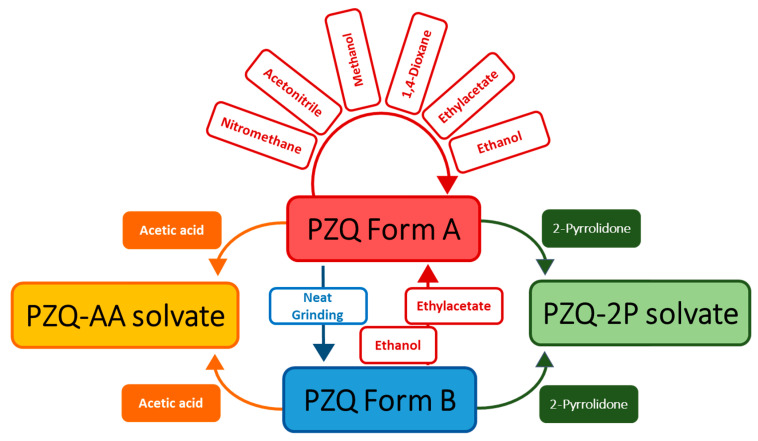
Sketched diagram of solid forms conversion by milling in the presence of different liquid additives.

As visible in Table 1 and Figure 2, the recurrent pattern of the solid recovered from the LAG experiments was Form A (these PXRD patterns are reported in Figure S1, with indication of the solvent used). Even changing the parameter η [23] (from 0.05 to 0.5 µL mg L^−1^), the collected form is structurally identical to the starting form.

Likewise, when starting from anhydrous Form B, the conversion from Form B to Form A was promoted. In this case milling with liquid additive (ethyl acetate and ethanol), led to polymorph interconversion, as already known for other substances [14] even if not documented yet for PZQ.

**Figure 3:**
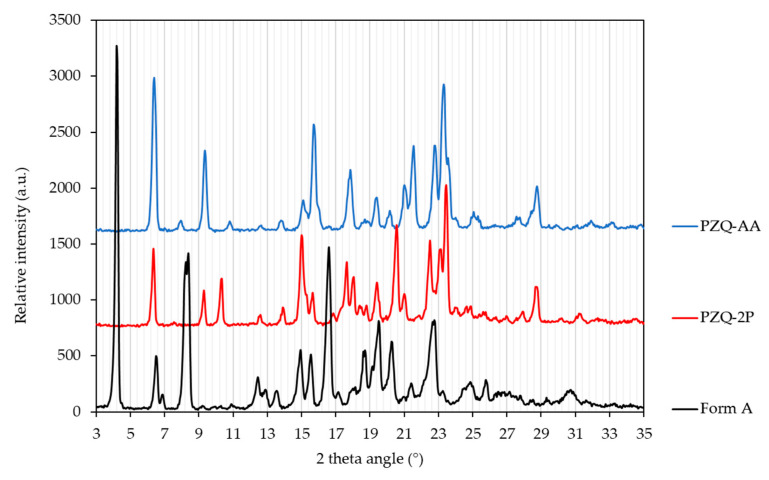
PXRD patterns of PZQ-AA (blue), PZQ-2P (red) and anhydrous Form A, commercially available (black) (wavelength: 1.5418 Å—in-house lab diffractometer).

PXRD patterns of the two obtained PZQ solvates obtained by grinding are shown in Figure 3, whilst the diffractograms of the solvates obtained by slurry procedure are reported in Figures S2 and S3. The diffraction peak positions of PZQ-AA and PZQ-2P are clearly different both from the known (starting) PZQ Form A, and from other known PZQ solid forms. However, the PXRD patterns of PZQ-AA and PZQ-2P are quite similar, which suggests that these two solvated forms should be isostructural [43]. The main reflections of PZQ-AA were at 6.21, 9.17, 14.90, 15.49, 17.69, 19.20, 20.85, 21.20, 22.57, 23.08 and 28.57° of 2θ. For PZQ-2P the main peaks were the following: 6.19, 9.11, 10.09, 14.85, 17.47, 17.83, 19.23, 20.30, 22.23, 22.87, 23.23 and 28.48° of 2θ (Figure 3).

**Figure 4:**
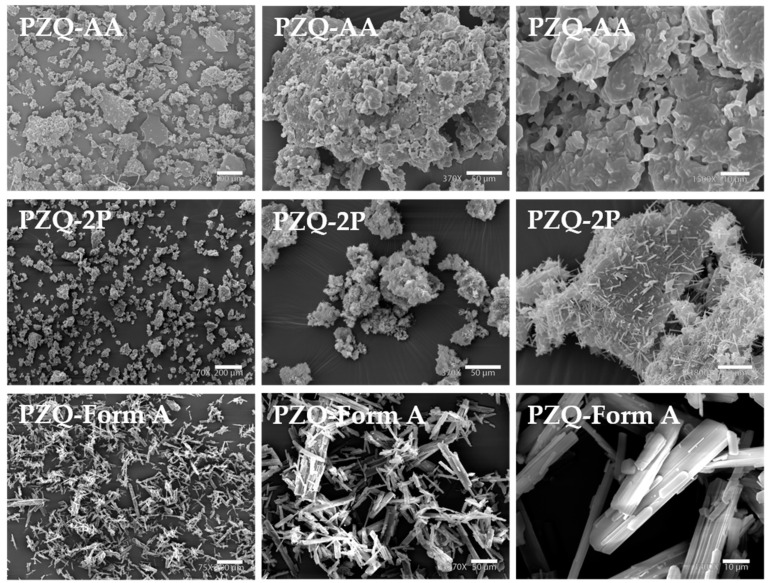
SEM images of samples obtained by mechanochemical synthesis: PZQ-AA (75×, **left**, 370×, **middle** and 1500×, **right**), PZQ-2P (70×, **left**, 370×, **middle** and 1800×, **right**) compared to raw PZQ Form A (75×, **left**, 270×, **middle**, and 1400×, **right**).

The two new solid forms exhibited some interesting differences in the habitus morphology (Figure 4). In the case of PZQ-AA SEM images showed layered blocks with almost flat particles, with a rough surface presenting some holes. Differently, in PZQ-2P the flat surface of the agglomerates is almost entirely covered by very thin needles somehow recalling the habitus of commercially available Form A, but much smaller in size.

**Figure 5:**
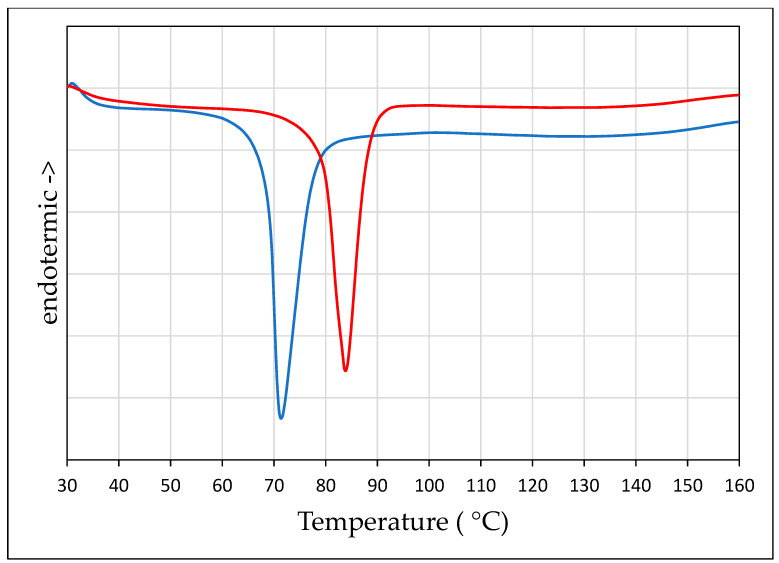
DSC of PZQ-AA (blue) and PZQ-2P (red).

The DSC curves of PZQ solvates are shown in Figure 5. It is noteworthy that each new phase was characterized by one single endothermic event in the considered temperature range, corresponding to the melting point at 72.26 °C (−74.15 J/g) for PZQ-AA and at 85.69 °C (−74.48 J/g) for PZQ-2P. At the Hot Stage Microscopy an analogous thermal behavior was noticed in both PZQ-AA and PZQ-2P cases (reported in Figure S4): at the temperature where the endotherm was seen at the DSC curve, the solid collapses forming a complete liquid phase. Recrystallization upon heating was evident starting from 88 °C with the appearance of thin needles, much smaller than original PZQ Form A crystal. The acicular crystals then melted in a temperature range compatible with the previously reported anhydrous Form B [9], and after that event no further crystallizations upon heating were noticed.

**Figure 6:**
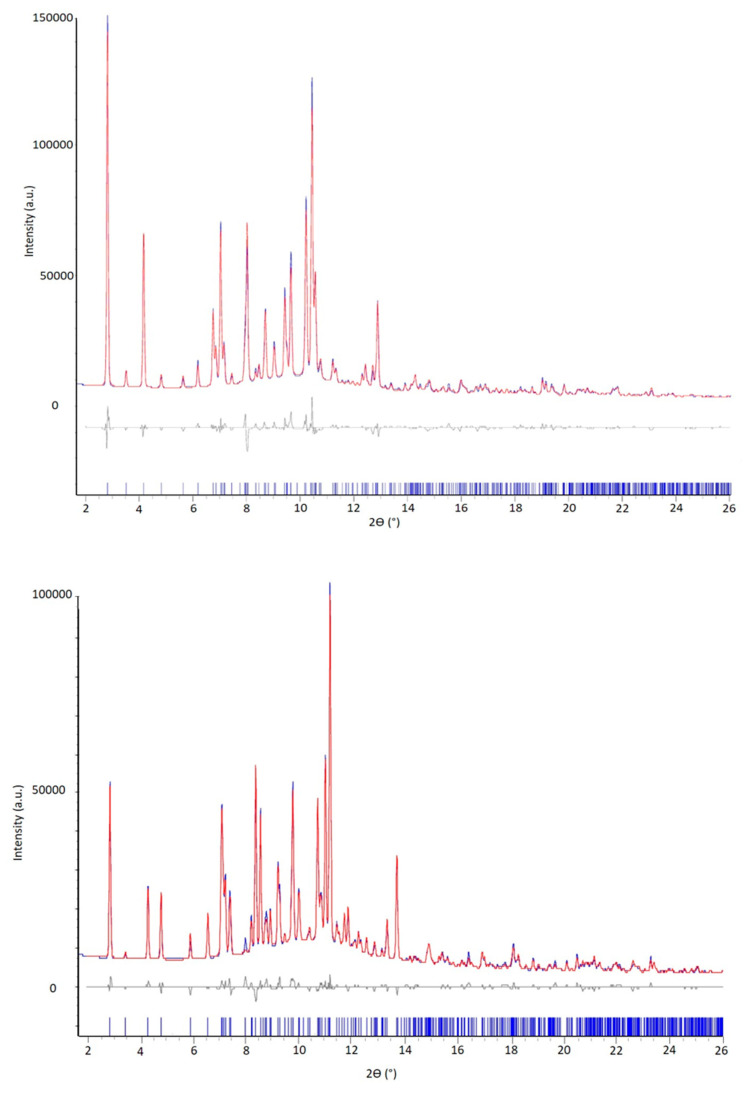
Rietveld refinement profile fit (obtained after the simulated annealing with EXPO14) of PZQ-AA (up) and PZQ-2P (bottom): in blue the experimental pattern (recorded using Synchrotron radiation, wavelength: 0.700 Å), in red the calculated one. The residuals are displayed on the bottom in gray and the reflection ticks in blue.

After these preliminary characterizations, to improve the signal-to-noise ratio at high 2θ angle the crystal structure of both solvated PZQ varieties was solved from the synchrotron X-ray powder diffraction data, presented in Figure 6. The higher crystallinity of these samples with respect to anhydrous forms previously prepared by neat grinding [9,10] along with the typical high signal to noise ratio allowed an undoubted indexing using the program EXPO2014 [28]. Both the samples belong to a P-1 triclinic unit cell with volume of 997.16 and 1058.04 Å^3^ for the PZQ-AA and PZQ-2P, respectively, very similar to the volume of the bis(Praziquantel) adipic acid called TELCAQ (CCDC 896766) [6,29].

The number of formula units per unit cell is Z = 2 and Z’ = 1, containing 4 molecules in the unit cell and 1 independent molecule of PZQ and 1 independent molecule of solvent in the asymmetric units, in agreement with SSNMR results (see below), with a reasonable density value of 1.240 g·cm^−3^. A first whole powder pattern fitting (Pawley method) on the experimental powder pattern resulted in a good Rwp of 5.36% and 4.07% for PZQ-AA and PZQ-2P, respectively. The refined unit cell parameters obtained for PZQ-AA and PZQ-2P are reported in Table 2. The solutions showing the best agreement with experimental data are shown in Figure 6. The final Rietveld refinement, where soft restrains on the atom distances (±0.03 Å) and angles (±0.1°) were applied, was performed in TOPAS V5 resulted in an R-Bragg factor of 3.32% and 2.58% for the PZQ-AA and PZQ-2P solvates, respectively. Notwithstanding the novel solvates show a triclinic unit cell with volume, cell parameters and crystal packed similar to the hemihydrate structure already published [11] the crystal packing comparison (see Figure S5) shows the main difference is due to the slippage of PZQ layers caused by the bulkiness of acetic acid and 2-pyrrolidone compared to water and to the hydrogen bond linkage between layers in the hemihydrate.

**Figure 7:**
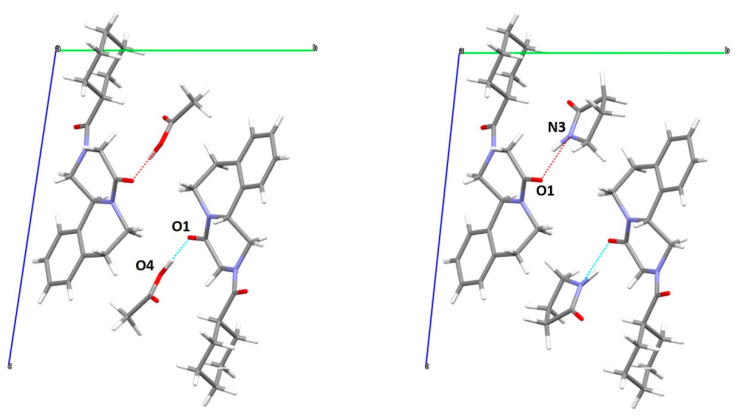
Capped-stick representation of the proposed structure of PZQ-AA (**left**) and PZQ-2P (**right**) viewed along a axis.

In both structures (Figure 7) the acetic acid and 2-pyrrolidone showed a strong interaction with the PZQ molecule via hydrogen bond, O1-O4 of 2.524 Å and O1-N3 of 2.760 Å for the PZQ-AA and PZQ-2P, respectively, as also attested by FT-IR analysis in both cases (see following Figure S6).

The FT-IR of PZQ-AA and PZQ-2P are reported in Figure S6: the presence of intermolecular hydrogen bonds between PZQ and the solvent molecules was displayed by the shift of various bands. In particular, a displacement toward lower wavenumbers of the carbonyl stretching vibrations of heterocyclic carbonyl (originally at 1651 cm^−1^ and observed at 1638 cm^−1^ in both PZQ-AA and PZQ-2P) may result from the decreased electron density of the carbonyl group when H-bonds are formed. Conversely, the changes in the carbonyl region cannot be inferred to conformational variation, due to the fact that both carbonyl groups are oriented in a *syn* conformation in both Form A and new solvates. Looking at the carbonyl stretching vibration of the solvents, in both solvates spectra, the pronounced ν(C=O) signals of the solvents are still noticeable and again downward shifted. A further diagnostic range in the spectrum is the 3500–3200 cm^−1^ interval [ν(N-H)], where a downward shift of the above-mentioned signal is visible in the solvate (originally present in the 2-pyrrolidone at 3488–3474 cm^−1^ and observed at 3243 cm^−1^ in PZQ-2P). This signal, absent in PZQ Form A, confirms the presence of 2-pyrrolidone in the structure. As for PZQ-AA, in the 3550–2500 cm^−1^ region, the broad bands of the OH- and CH-vibrations is very complex: only two signals ranging about 2600 cm^−1^ are undoubtedly attributable to acetic acid in the solvate spectrum.

The SSNMR analysis was instrumental in assisting the structure solution from powder data, checking the reliability of the solved structure in combination with DFT calculations [44,45] and evaluating possible hydrogen bond interaction between PZQ and the solvent molecules. Indeed, it is well known that in the case of molecular crystals, SSNMR can provide several information such as number of independent molecules per unit cell [46], presence of solvent molecules in the crystal and assessment of the stoichiometry [47], identification of different crystal forms [46,47], evaluation of hydrogen bond interactions and network [48,49].

In the case of the samples under investigation, the SSNMR analyses highlighted the marked differences between the two solvates, in addition to those with original PZQ Form A and B. The average full width at half maximum (FWHM) value for the signals indicates a low degree of crystallinity of the samples (FWHM ~ 140 and 150 Hz for PZQ-AA and PZQ-2P, respectively) in agreement with the typical limited crystallinity of a sample obtained by grinding in comparison to those obtained by solution crystallization. Figure S7 reports the ^13^C CPMAS SSNMR spectra of PZQ-AA and PZQ-2P, with the assignments of the relevant resonances, together with those of PZQ Form A and B used as a comparison. Table 3 reports experimental and computed ^13^C chemical shifts with assignments for both PZQ-AA and PZQ-2P. The presence of the solvent molecules can be easily detected by the peaks at 172.1 (COOH) and 19.7 (CH_3_) ppm for PZQ-AA and at 177.5 (C=O) and 19.4, 28.9 and 40.4 (CH_2_) ppm for PZQ-2P. In the case of PZQ-AA, one of the C=O signal is not visible, due to the overlapping with the COOH signal of acetic acid (172.1 ppm), while in the case of PZQ-2P the additional C=O is easily recognized. In both spectra, the number of signals is consistent with the presence of one PZQ and one solvent molecule in the asymmetric unit. Despite the intrinsic quantitative limitations of the CPMAS experiment, an accurate integration of the signals confirms the 1:1 stoichiometry for both solvates. Indeed, since we are comparing the same group (C=O) with similar limited mobility, we can safely suppose nearly equal cross-polarization rates (i.e., T_IS_) and ^1^H T_1ρ_ values. Similar, the approach was successfully tested for other systems [50]. The position of the C=O C4 signal, at higher frequencies in PZQ-AA (166.8 ppm) and PZQ-2P (165.7 ppm) with respect to that of PZQ Form B (164.3) may be taken as evidence of their involvement in intermolecular hydrogen bonding with the solvent molecules [51].

The calculated NMR parameters show excellent agreement with the experimental ones (Figures S8 and S9 and Table 3). The disagreement between the calculated and experimental ^13^C NMR signal for the carboxylic carbon of PZQ-AA may be attributed to the general difficulty encountered when modelling electron distribution around the functional groups with ionizable protons [52] using semi-local DFT functionals such as PBE. In addition, comparison of the calculated and experimental NMR shifts by means of RMS error once again revealed excellent agreement (RMSE = 2.09 and 1.83 ppm). These data definitely confirm the reliability of the structure solved from PXRD data.

**Figure 8:**
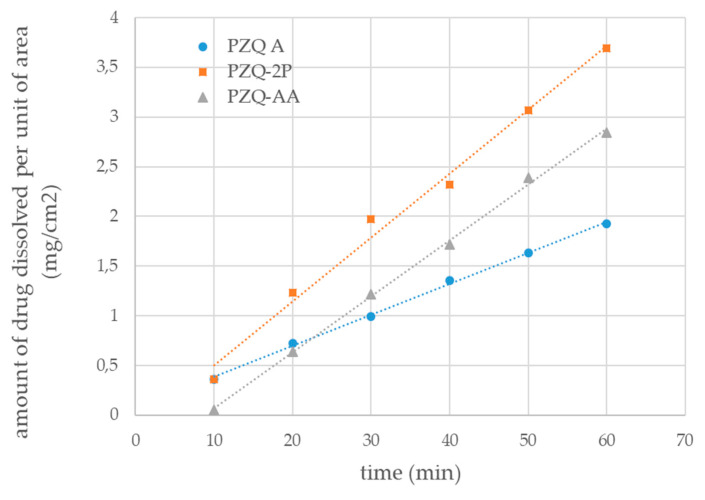
Intrinsic dissolution profiles of PZQ-AA (grey triangles), PZQ-2P (orange squares) and raw PZQ Form A (blue circles) (mean of triplicated measurements); linear regression trend for each profile is represented as dotted lines.

The water solubility (after 48 h at 25 °C) and the intrinsic dissolution rate were also carried out for the new solid forms and compared to anhydrous PZQ Form A. PZQ-AA reached a concentration of 311.91 ± 15.22 mg/L after 48 h while for PZQ-2P was 340.27 ± 23.18 mg/L, both far superior to that reported at the same temperature for Form A [10]. This is in agreement with commonly described behavior of the solvated form to be more soluble than the corresponding anhydrous form [12]. Proportionally to the solubility, the intrinsic dissolution rate in water at 37 °C was enhanced in the new forms as reported in Figure 8: values of 0.0563 ±0.0013 and 0.0640 ± 0.0072 mg/cm^2^/min were recorded for PZQ-AA and PZQ-2P, respectively, about double than that of PZQ Form A (0.0312 ± 0.00283 mg/cm^2^/min). The statistical comparison between the PZQ-AA and PZQ-2P value did not reveal any significant difference, which was conversely detected when comparing the IDR of both solvates with the one of Form A.

Finally, the physical stability of the obtained monosolvated forms kept at ambient temperature in sealed vials was checked by PXRD. Both PZQ-AA and PZQ-2P exhibited a very noticeable physical stability of at least 18 months (as visible from Figures S2 and S3, respectively). Additionally, mechanical treatment at 25 Hz for 200 min without interruptions was almost ineffective on both of the two solvates, since the same PXRD patterns were collected. Conversely, when PZQ-AA was heated at a constant temperature of 50 °C under vacuum over-night, the obtained product, as assessed by means of PXRD, changed dramatically and was a mixture of unidentified phases. As for PZQ-2P, the same pattern as the fresh sample was obtained, attesting its greater stability, also probably related to the higher boiling point of 2-pyrrolidone.

## 4. Discussion

The crystal form screening, conducted by mechanochemistry in the presence of eight commonly used solvents, gave crystalline solvates only in the case of acetic acid and 2-pyrrolidone, even though top solvate-forming solvents (e.g., methanol and acetonitrile) [12] were present among the screened additives.

Since discriminative solvate formation occurs with the solvate selectivity based on the solvent’s functionality to provide strong intermolecular interactions, the properties of the liquid additives were hence examined to understand the reason of the selective formation of solvates of PZQ. Main physicochemical properties of the solvents, as documented in literature [39,40,41,42,53], are listed in Table 1.

The failure of solvate outcome is often attributed to the evaporation of the volatile liquid during the milling process. However, this was not the case here, due to the very short milling time (30 min), the observed milling temperature (about 30 °C, see Section 2.2) largely inferior to the boiling temperatures of the used solvents. Further, the forming-solvate acetic acid has a boiling temperature very similar to many other liquid additives in the list. Indeed, methanol capability to form solvates by grinding has been previously documented despite its volatility [54,55]; however, it is not able to form a solvate with praziquantel. This further confirms that the selectivity of solvate formation of praziquantel in presence of acetic acid and 2-pyrrolidone is not due to their boiling point.

A commonly recognized important parameter for formation of solvates is the solid solubility in the solvents. While its importance in solution crystallization is generally recognized, it is not so in mechanochemical reaction in the presence of liquid additives, about which contradictory opinions can be found in the literature [16,56,57]. Indeed, during LAG synthesis, high degrees of supersaturation are always present since only small amounts of solvent are used, therefore increasing the role of other variables. Additionally, in this case, comparing PZQ solubility in different media from literature [53,58,59] with our results, and remembering that PZQ is able to yield a hemihydrate in water where is sparingly soluble [24], a relationship between solid solubility and solvate formation cannot be proven.

In the PZQ molecule, only the oxygen atoms of the amide group can function as hydrogen bond acceptors because the free electron pair of the nitrogen atoms are involved in π electron delocalization. Additionally, there are three types of C-H acidic hydrogen atoms for the generation of attractive secondary C-H···O contacts [6]. Further, PZQ has a documented potential to form multicomponent crystals (e.g., cocrystals or hydrates) with reagents containing hydrogen bond donors [6,11]. As mentioned in the literature, the strength of H-bonding between the solvent and the solute molecules can be evaluated through the solvent properties, and the values of α and β, respectively, can be used to evaluate the H-bond donation ability and H-bond acceptance ability or electron pair donation ability to form a coordinative bond [40]. As visible from Table 1, the eight studied solvents are characterized by variable properties. However, the solvates were only obtained from acetic acid and 2-pyrrolidone. By comparing the parameters of the eight solvents, acetic acid has the highest alpha value, and the lowest cohesive energy density (CED). Alpha parameter reflects a good H-bond donor propensity, whereas CED reveals the strength of solvent-solvent interactions and hence is involved in the solute-solvent interactions. These data can therefore be evoked as the main responsible of the formation of the PZQ-AA. As for 2-pyrrolidone, in addition to the H-bond donation to the amide group oxygen atom (note that the solvent has intermediate β value in the list), also the attractive secondary C-H···O contacts might be involved: this would be in agreement with its highest documented β parameter. Further, the very high cohesive energy density value of 2-pyrrolidone might not play a role which was conversely suggested previously in acetic acid case. Finally, π parameter, a further classical parameter evoked in solvate formation context [60] describing a combination of polarity and polarizability, did not show a correlation with our results. In conclusion, from our survey it can be speculated that PZQ solvate formation is not simply dependent on any individual solvent parameter but on the combined impact of solvent hydrogen bond-donor propensities and cohesive energy density.

Examining the molecular structure of the newly synthetized PZQ-AA and PZQ-2P, a *syn* conformation of the C=O groups of the piperazinone-cyclohexylcarbonyl segment is adopted. Hence, in contrast to known solvated forms (single enantiomers or racemic PZQ hemihydrates [6,7,11] providing an *anti*-conformation of the C=O groups, in the two newly synthetized solvates these groups retain the same stereochemistry as the starting PZQ Form A. This feature is peculiar and rather counterintuitive, due to the fact that the *anti*-orientation would provide a larger spatial separation of the C=O groups and might enable in principle an easier insertion of the complementary hydrogen bond forming functions of solvent molecules. In fact, PZQ has a documented higher propensity to interact via hydrogen bonds when in an *anti*-conformation [6]. This stereochemistry was also the most common in the case of PZQ cocrystals with dicarboxylic acids, cited in previous structural reports [6]. Noteworthy when grinding process started from PZQ Form B (also featuring the *anti*-conformation), PZQ is switched as well to the *anti*-antagonism.

However, considering the *syn* conformation also in the light of the above-mentioned H-bond interactions, it can be hypothesized that this stereochemistry is more favorable for the insurgence of secondary attractive H-bonds, in particular in relation to the cyclohexylcarbonyl moiety, where—as a matter of fact—the relatively rigid PZQ molecular structure has its limited conformational flexibility.

Of note is the fact that, contrary to what reported for PZQ hemihydrate [11], the formation of the new solvates depends on the solvent used in the course of grinding rather than on the starting solid form of PZQ. In particular, differently from the hemihydrate case, is not mandatory to yield the solvates an intermediate amorphous/metastable state, but a direct grinding process in the presence of liquid additive is adequate, provided that the suitable liquid additive is present. The retention of the original carbonyl stereochemistry in both the solvates might be an additional reason.

Interestingly, this pair of newly discovered solvates belong to the class of isostructural solvates [12,61,62,63] since structures belong to the same space group (P-1) and display similar unit-cell dimensions (with only small distortions) and conformations, as well as isostructural crystal packings. The conformation of the core molecule seems predominantly responsible for governing the isostructurality. According to this, very similar melting enthalpies at the DSC and Intrinsic Dissolution Rate values were observed in the two solvates, and analogous physical stability was observed. In this latter regard, the results are not surprising, since it is known that the liquid insertion in a crystal lattice, even though it can sometimes bring some disorder in the structure, can also favor the formation of hydrogen bonds and strong interactions that improve the stability of the system, which is noticeably remarkable.

In terms of water solubility and IDR, PZQ-AA and PZQ-2P showed the expected favorable biopharmaceutical performance, which is superior to that of anhydrous Form A. The most usual explanation of such performance is that the negative Gibbs free energy of mixing of the organic solvent, released during the dissolution of a solvate, contributes to the Gibbs free energy of solution, increasing the thermodynamic driving force for the dissolution process [12]. In other words, this is due to the fact that, a non-aqueous solvate phase could be considered as being a high-energy form of the solid with respect to dissolution in water. As previously said, since the two solvates are nearly isomorphic, it is not surprising that the biopharmaceutical performance of the two solvates are very similar.

## 5. Conclusions

The propensity of solvate formation of PZQ was studied by mechanochemical screening using eight selected solvents. Such screening provided two new solvates, both with 1:1 host-guest stoichiometry, with acetic acid and 2-pyrrolidone. The crystal structures were solved from synchrotron X-ray powder diffraction data: the solvates being isostructural, unit cell being triclinic. In both structures the acetic acid and 2-pyrrolidone showed a strong interaction with the PZQ molecule via hydrogen bond, providing a physical stability of at least 18 months.

Mechanochemical approach has demonstrated also in this case to be a viable process for discovering new crystal forms. Although the screening was limited to eight liquids, these analyses provided important findings for hypothesizing the possible mechanism of interaction between PZQ and the liquid additives, giving reason for the peculiar behavior in the presence of acetic acid and 2-pyrrolidone. For the formation of these PZQ solvates, differently from previous PZQ hemihydrate, the type of solvent and its capacity of interaction with the solid affect the course of the reaction rather than the identity of PZQ crystal form used as starting material.

As PZQ is a drug substance whose absorption is determined by the dissolution rate, the demonstration of the existence of solvates, in addition to anhydrous polymorphs and hydrates, highlights the need for proper survey of PZQ solid forms landscape and a careful consideration during manufacture and formulation of experimental conditions that could be associated with the insurgence of unpredicted drug crystal forms. Further, the effect of liquid additives in mechanochemistry requires more systematic work and the interplay of various scientific disciplines in order to draw the ultimate picture of solvate formation.

## Figures and Tables

**Table 1:** Physicochemical parameters of the solvents and observed Praziquantel solid forms via grinding in the presence of each liquid additive.

Solvent	Boiling Point (°C) [39]	H donor Ability (α) [40]	H bond Acceptor Ability (β) [40]	Polarizability [40]	Cohesive Energy Density (J mol/mL) [41]	Starting PZQ Solid Form	Observed Solid Form
acetic acid	118	112	45	64	370.80	A	AA
						B	AA
acetonitrile	81.6	19	40	75	522.95	A	A
1,4-dioxane	101.3	0	37	55	372.17	A	A
ethanol	78.3	86	75	54	618.87	A	A
						B	A
ethyl acetate	77.1	0	45	55	300.64	A	A
						B	A
methanol	64.7	98	66	60	808.26	A	A
nitromethane	101.0	22	06	85	587.22	A	A
2-pyrrolidone	245.0	36	77	85	789.61 [42]	A	2P
						B	2P

**Table 2:** Crystallographic data for PZQ solvates (refined unit cell parameters at ambient temperature).

Phase	PZQ-AA	PZQ-2P
Empirical Formula	C_19_H_24_N2O_2_·(C_2_H_4_O_2_)	C_19_H_24_N2O_2_·(C_4_H_7_NO)
Formula weight	372.46	397.51
Crystal system	triclinic	Triclinic
Space group	P-1	P-1
a (Å)	6.0762 (2)	6.1839 (2)
b (Å)	11.6197 (4)	12.2615 (3)
c (Å)	14.5619 (8)	14.5539 (5)
Volume (Å^3^)	997.16	1058.04
α (°)	97.292 (3)	93.823 (2)
β (°)	98.851 (3)	100.064 (2)
γ (°)	96.987 (2)	101.693(1)
Density (g·cm^−3^)	1.240	1.248
Z	2	2
R-Bragg factor (%)	3.32	2.58
wR factor (%)	4.70	4.04
Goodness of fit	4.65	3.79

**Table 3:** Experimental and computed ^13^C chemical shifts (ppm) with assignments for PZQ-AA and PZQ-2P.

Atom	Group	PZQ-AA		PZQ-2P	
		δ_iso_(exp)/ppm	δ_iso_(calc)/ppm *	δiso(exp)/ppm	δiso(calc)/ppm *
7’	C=O	172.1	172.68	172.9	173.22
4	C=O	166.8	167.92	165.7	166.66
11a	C_q_	133.1	135.96	133.4	135.75
7a	C_q_	134.8	138.03	133.4	137.14
8	CH_(ar.)_	129.1	132.30	128.1	131.17
11	CH_(ar.)_	124.7	126.84	124.6	126.89
10	CH_(ar.)_	126.7	128.66	128.1	130.88
9	CH_(ar.)_	126.7	128.91	127.4	130.10
11b	CH_(aliph.)_	53.7	53.42	54.0	54.2
3	CH_2_	46.4	45.51	47.2	46.3
1	CH_2_	43.3	41.07	44.3	42.80
1’	CH_(aliph.)_	39.8	39.06	39.7	39.63
6	CH_2_	37.2	34.71	37.6	34.84
6’	CH_2_	28.9	27.68	28.9	27.22
2’	CH_2_	28.9	27.38	28.9	28.41
7	CH_2_	26.4	25.30	27.8	26.22
4’	CH_2_	27.0	25.61	25.8	25.05
3’	CH_2_	26.4	25.46	25.8	24.37
5’	CH_2_	25.0	23.60	25.8	24.58
AA **	COOH	172.1	177.13		
AA	CH_3_	19.7	18.03		
2P	CH_2_			19.4	19.09
2P	CH_2_			28.9	28.16
2P	CH_2_			40.4	41.48
2P	CONH			177.5	178.94

* The calculated chemical shieldings are reported in Table S1. ** AA and 2P mean acetic acid and 2-pyrrolidone, respectively.

## Data Availability

Not applicable.

## Supplementary Materials

The following are available online at https://www.mdpi.com/article/10.3390/pharmaceutics13101606/s1. Figure S1. PXRD pattern of the solids retrieved after grinding PZQ A and B in presence of different liquid additives and PZQ A as a reference. Figure S2. PZQ-AA: fresh sample, after 18 months at room temperature *, under vacuum overnight at 50 °C *, ground for 200 min at 25 Hz * and PZQ-AA prepared by slurry method. Figure S3. PZQ-2P: fresh sample, after 18 months at room temperature*, under vacuum overnight at 50 °C *, ground for 200 min at 25 Hz * and PZQ-AA prepared by slurry method. Figure S4. Optical microscopy images of PZQ-AA and PZQ-2P upon heating (with indication of operating temperature in each image) (Magnification 100×). Figure S5: Crystal packing comparison along axis of (a) PZQ-AA, (b) PZQ-2P and (c) PZQ hemihydrate form. Figure S6: FT-IR spectra of PZQ-AA (blue), PZQ-2P (red) and raw PZQ Form A (green). Frames highlight diagnostic regions with above-mentioned different colors. Figure S7. 13C (100.65 MHz) CPMAS SSNMR spectra of (a) PZQ Form A, (b) PZQ Form B, (c) PZQ-2P, and (d) PZQ-AA acquired at 12 kHz. Relevant assignments are reported for the PZQ molecule (b) and for the solvent molecules (c,d) resonances. Figure S8. Comparison of experimental and calculated ^13^C SSNMR spectra of PZQ-AA (top) and PZQ-2P (bottom). The only significant disagreement is found for the COOH signal, at 177.1 and 172.1 ppm in the PZQ-AA calculated and experimental spectrum, respectively. The 5 ppm difference may be attributed to the difficulty in modelling the electronic environment around an acidic ionizable proton. Figure S9. Quantitative comparison of experimental and calculated ^13^C SSNMR spectra of PZQ-AA (top) and PZQ-2P (bottom). Table S1 Calculated shift/ppm and calculate shielding/ppm (for atom numbering please refer to Scheme). Externally hosted supplementary file S1: Link: http://www.ccdc.cam.ac.uk/structuresDescription (Accessed on 27 September 2021): CCDC 2074789 and CCDC 2074793 contain the supplementary crystallographic data for Praziquantel acetic acid monosolvate (PZQ-AA) and Praziquantel 2-pyrrolidone monosolvate (PZQ-2P).
