# Supplementary material for: Mechanochemical Synthesis and Physicochemical Characterization of Previously Unreported Praziquantel Solvates with 2-Pyrrolidone and Acetic Acid
Source: Pharmaceutics. 2021 Oct 2;13(10):1606. doi: 10.3390/pharmaceutics13101606 (PMC8540171; doi:10.3390/pharmaceutics13101606)

# checkCIF/PLATON report

You have not supplied any structure factors. As a result the full set of tests cannot be run.

THIS REPORT IS FOR GUIDANCE ONLY. IF USED AS PART OF A REVIEW PROCEDURE FOR PUBLICATION, IT SHOULD NOT REPLACE THE EXPERTISE OF AN EXPERIENCED CRYSTALLOGRAPHIC REFEREE.

No syntax errors found. CIF dictionary Interpreting this report

## Datablock: PZQ_2P

---

```
Bond precision:  C-C = 0.0006 A          Wavelength=0.70000

Cell:           a=6.184(2)        b=12.2615(3)      c=14.5539(5)
                alpha=93.823(2)   beta=100.064(2)   gamma=101.693(1)
Temperature:    298 K

                Calculated                Reported
Volume          1058.0(3)                 1058.02(5)
Space group     P -1                      P-1
Hall group      -P 1                      -P 1
Moiety formula  C19 H24 N2 O2, C4 H7 N O  C19 H24 N2 O2, C4 H7 N O
Sum formula     C23 H31 N3 O3             C23 H31 N3 O3
Mr              397.51                    397.51
Dx,g cm-3       1.248                     1.248
Z               2                         2
Mu (mm-1)       0.080                     0.080
F000            428.0                     428.0
F000'           428.16
h,k,lmax        3,7,9
Nref            581
Tmin,Tmax
Tmin'

Correction method= Not given

Data completeness= 0.000          Theta(max)=

R(reflections)=                   wR2(reflections)=

S =                          Npar=
```

---

The following ALERTS were generated. Each ALERT has the format
**test-name_ALERT_alert-type_alert-level**.
Click on the hyperlinks for more details of the test.

---

## Alert level C

```
PLAT351_ALERT_3_C Long   C-H (X0.96,N1.08A)  C22      - H29      .       1.11 Ang.
PLAT353_ALERT_3_C Long   N-H (N0.87,N1.01A)  N3       - H31      .       1.02 Ang.
```

## Alert level G

```
ABSMU01_ALERT_1_G  Calculation of _exptl_absorpt_correction_mu
                ns not performed for this radiation type.
PLAT007_ALERT_5_G Number of Unrefined Donor-H Atoms ..............         1 Report
PLAT092_ALERT_4_G Check: Wavelength Given is not Cu,Ga,Mo,Ag,In Ka    0.70000 Ang.
PLAT152_ALERT_1_G The Supplied and Calc. Volume s.u. Differ by ...         25 Units
PLAT793_ALERT_4_G Model has Chirality at C3          (Centro SPGR)          S Verify
PLAT949_ALERT_5_G Unusual Experimental Reflection Sigma(I)'s .....     Please Check
```

```
   0 ALERT level A = Most likely a serious problem - resolve or explain
   0 ALERT level B = A potentially serious problem, consider carefully
   2 ALERT level C = Check. Ensure it is not caused by an omission or oversight
   6 ALERT level G = General information/check it is not something unexpected

   2 ALERT type 1 CIF construction/syntax error, inconsistent or missing data
   0 ALERT type 2 Indicator that the structure model may be wrong or deficient
   2 ALERT type 3 Indicator that the structure quality may be low
   2 ALERT type 4 Improvement, methodology, query or suggestion
   2 ALERT type 5 Informative message, check
```

It is advisable to attempt to resolve as many as possible of the alerts in all categories. Often the minor alerts point to easily fixed oversights, errors and omissions in your CIF or refinement strategy, so attention to these fine details can be worthwhile. In order to resolve some of the more serious problems it may be necessary to carry out additional measurements or structure refinements. However, the purpose of your study may justify the reported deviations and the more serious of these should normally be commented upon in the discussion or experimental section of a paper or in the "special_details" fields of the CIF. checkCIF was carefully designed to identify outliers and unusual parameters, but every test has its limitations and alerts that are not important in a particular case may appear. Conversely, the absence of alerts does not guarantee there are no aspects of the results needing attention. It is up to the individual to critically assess their own results and, if necessary, seek expert advice.

**Publication of your CIF in IUCr journals**

A basic structural check has been run on your CIF. These basic checks will be run on all CIFs submitted for publication in IUCr journals (*Acta Crystallographica*, *Journal of Applied Crystallography*, *Journal of Synchrotron Radiation*); however, if you intend to submit to *Acta Crystallographica Section C* or *E* or *IUCrData*, you should make sure that full publication checks are run on the final version of your CIF prior to submission.

**Publication of your CIF in other journals**

Please refer to the *Notes for Authors* of the relevant journal for any special instructions relating to CIF submission.

## PLATON version of 22/03/2021; check.def file version of 19/03/2021

**Datablock PZQ_2P** - ellipsoid plot

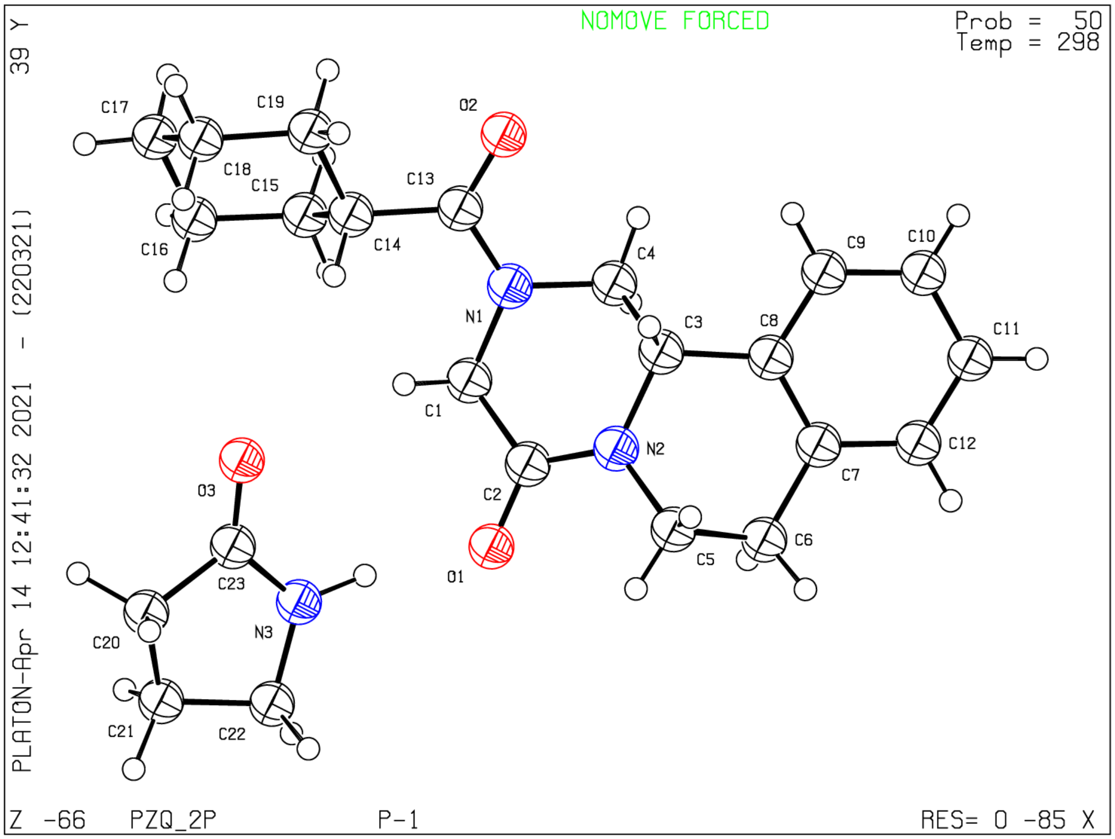

Supplement: Supplementary file 1 [file pharmaceutics-13-01606-s001.zip › checkcif-2P-final.pdf]
