# Supplementary material for: Mechanochemical Synthesis and Physicochemical Characterization of Previously Unreported Praziquantel Solvates with 2-Pyrrolidone and Acetic Acid
Source: Pharmaceutics. 2021 Oct 2;13(10):1606. doi: 10.3390/pharmaceutics13101606 (PMC8540171; doi:10.3390/pharmaceutics13101606)
Supplement: Supplementary file 1 [file pharmaceutics-13-01606-s001.zip › pharmaceutics-1373176-supplementary.pdf]

pharmaceutics

MDPI

# Supplementary Materials: Mechanochemical Synthesis and Physicochemical Characterization of Previously Unreported Praziquantel Solvates with 2-Pyrrolidone and Acetic Acid

**Debora Zanolla, Lara Gigli, Dritan Hasa, Michele Remo Chierotti, Mihails Arhangelskis, Nicola Demitri, William Jones, Dario Voinovich and Beatrice Perissutti**

**Supplementary information**

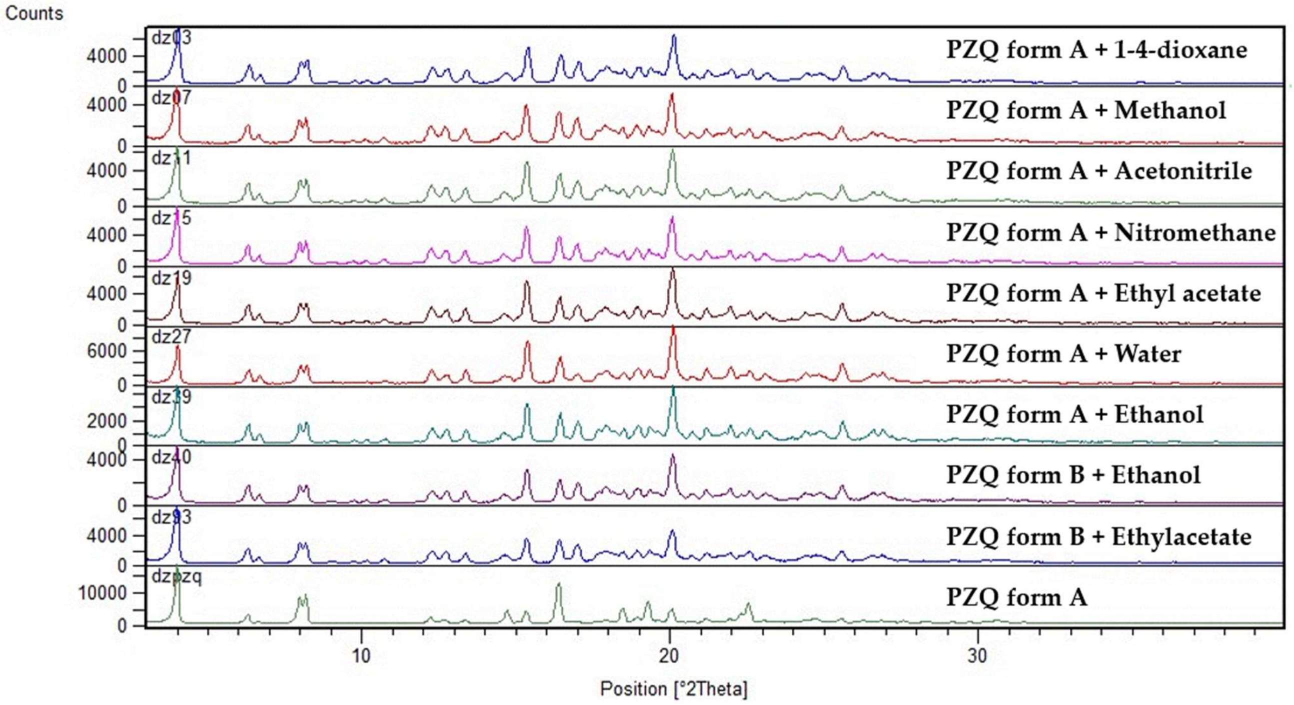


**Figure S1.** PXRD pattern of the solids retrieved after grinding PZQ A and B in presence of different liquid additives and PZQ A as a reference.

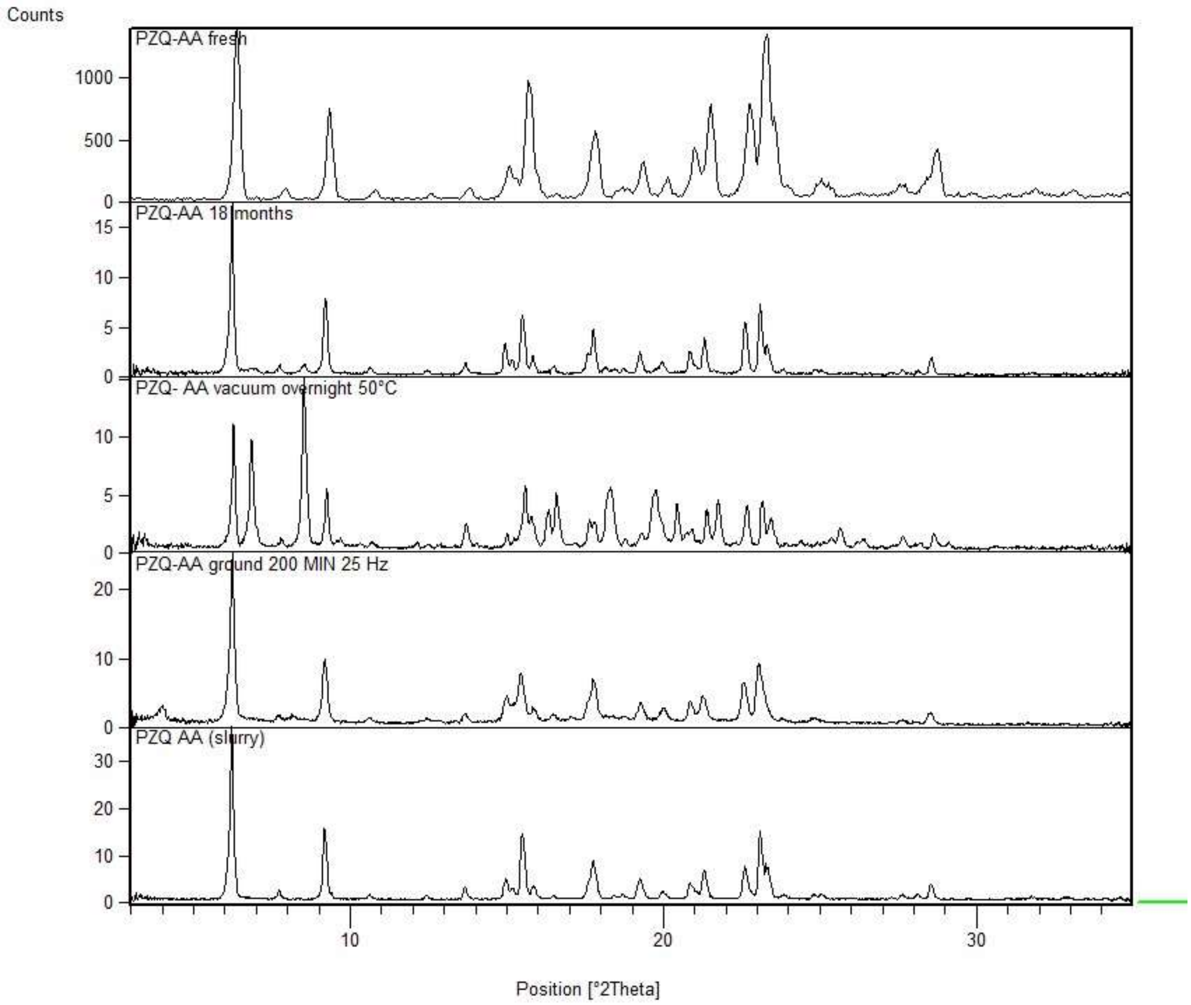


**Figure S2.** PZQ-AA: fresh sample, after 18 months at room temperature*, under vacuum overnight at 50°C*, ground for 200 minutes at 25 Hz* and PZQ-AA prepared by slurry method*.( *=PXRD pattern were collected using a D2 Phaser(Bruker), using a zero background (Sylicon) sample holder, from 3 to 35° of 2θ, 0.02° step, time/step 0.6 sec, at 30 V, 10 mA).

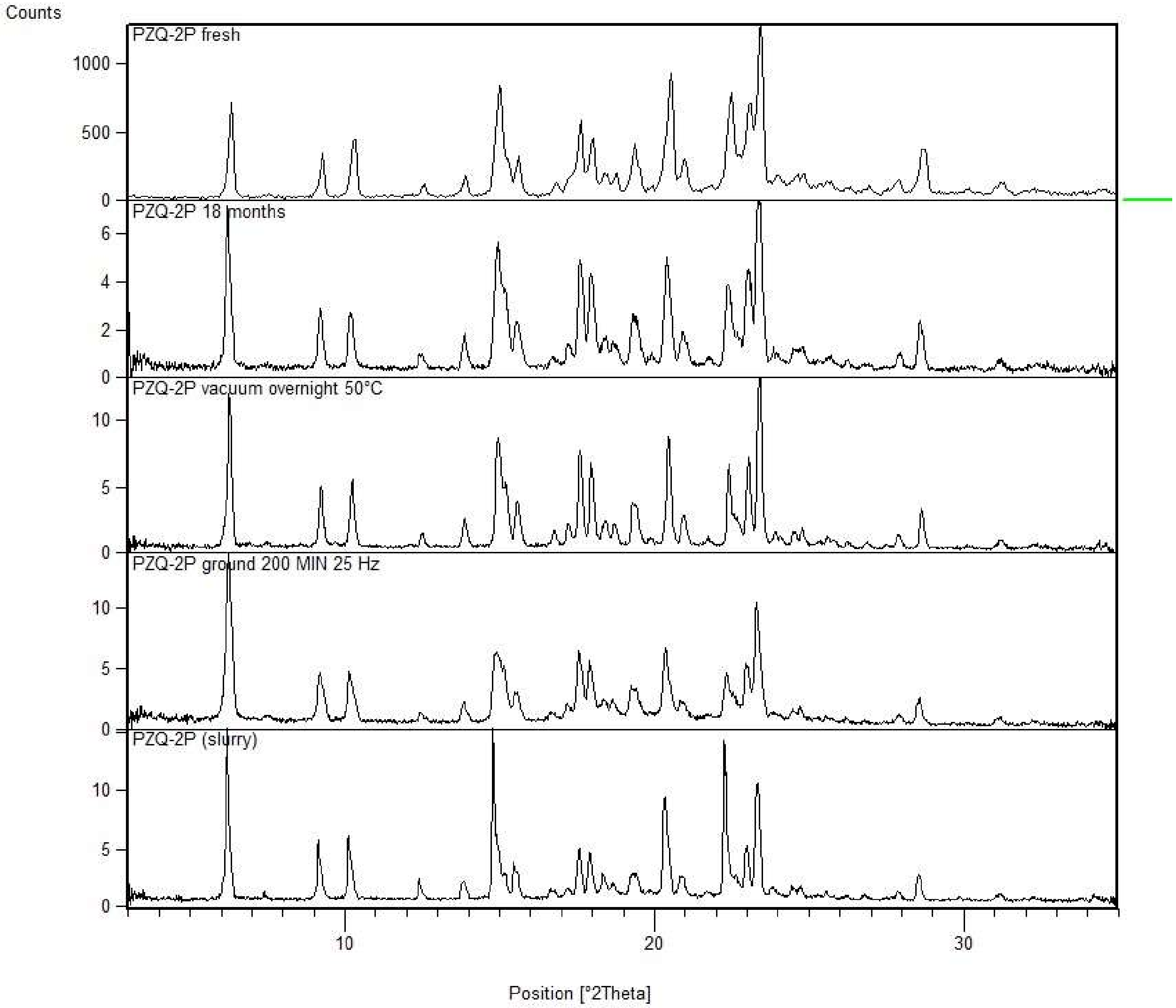


**Figure S3.** PZQ-2P: fresh sample, after 18 months at room temperature*, under vacuum overnight at 50°C*, ground for 200 minutes at 25 Hz* and PZQ-AA prepared by slurry method*.( *=PXRD pattern were collected using a D2 Phaser (Bruker), using a zero background (Sylicon) sample holder, from 3 to 35° of 2θ, 0.02° step, time/step 0.6 sec, at 30 V, 10 mA)

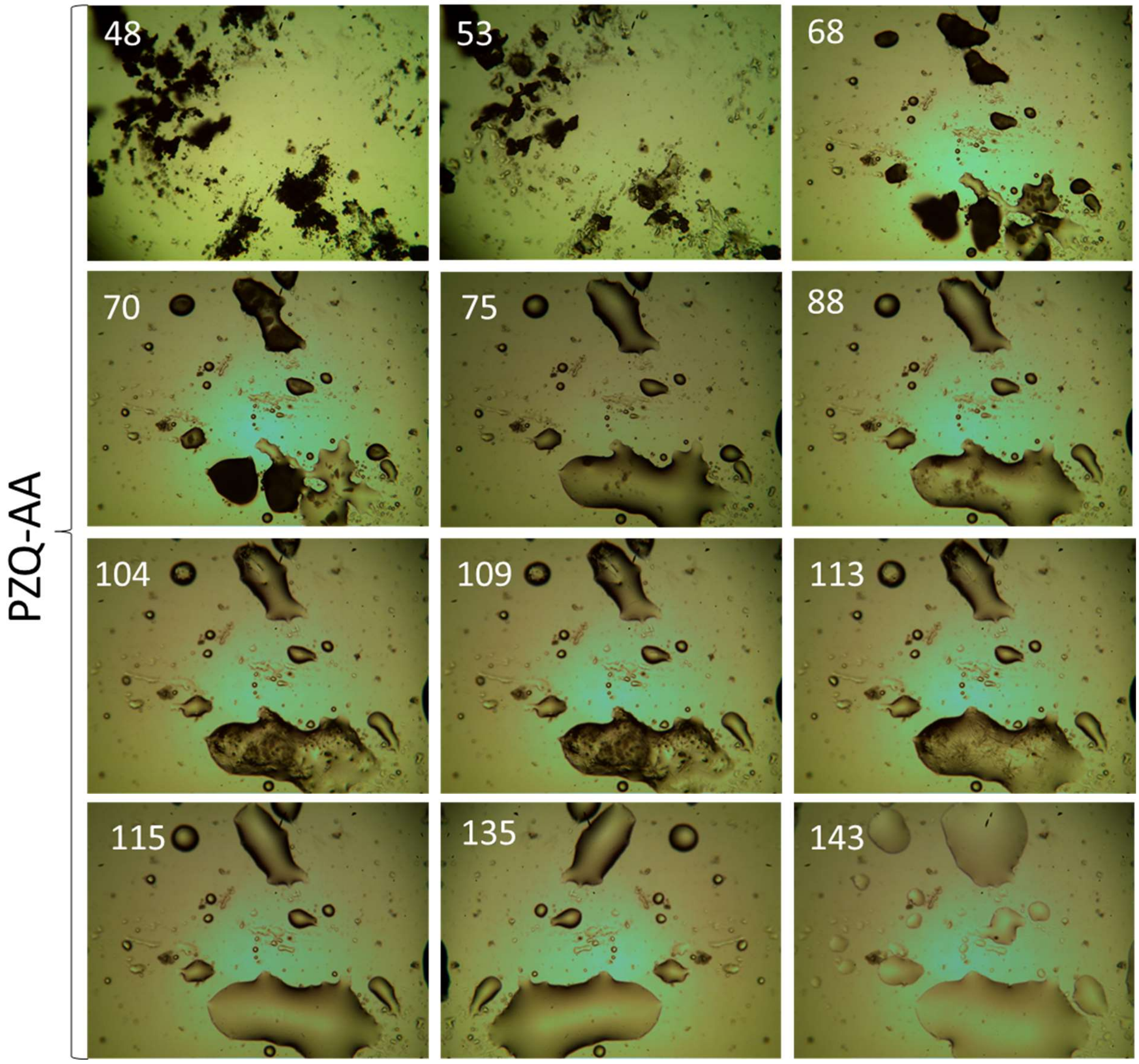
PZQ-AA
48
53
68
70
75
88
104
109
113
115
135
143

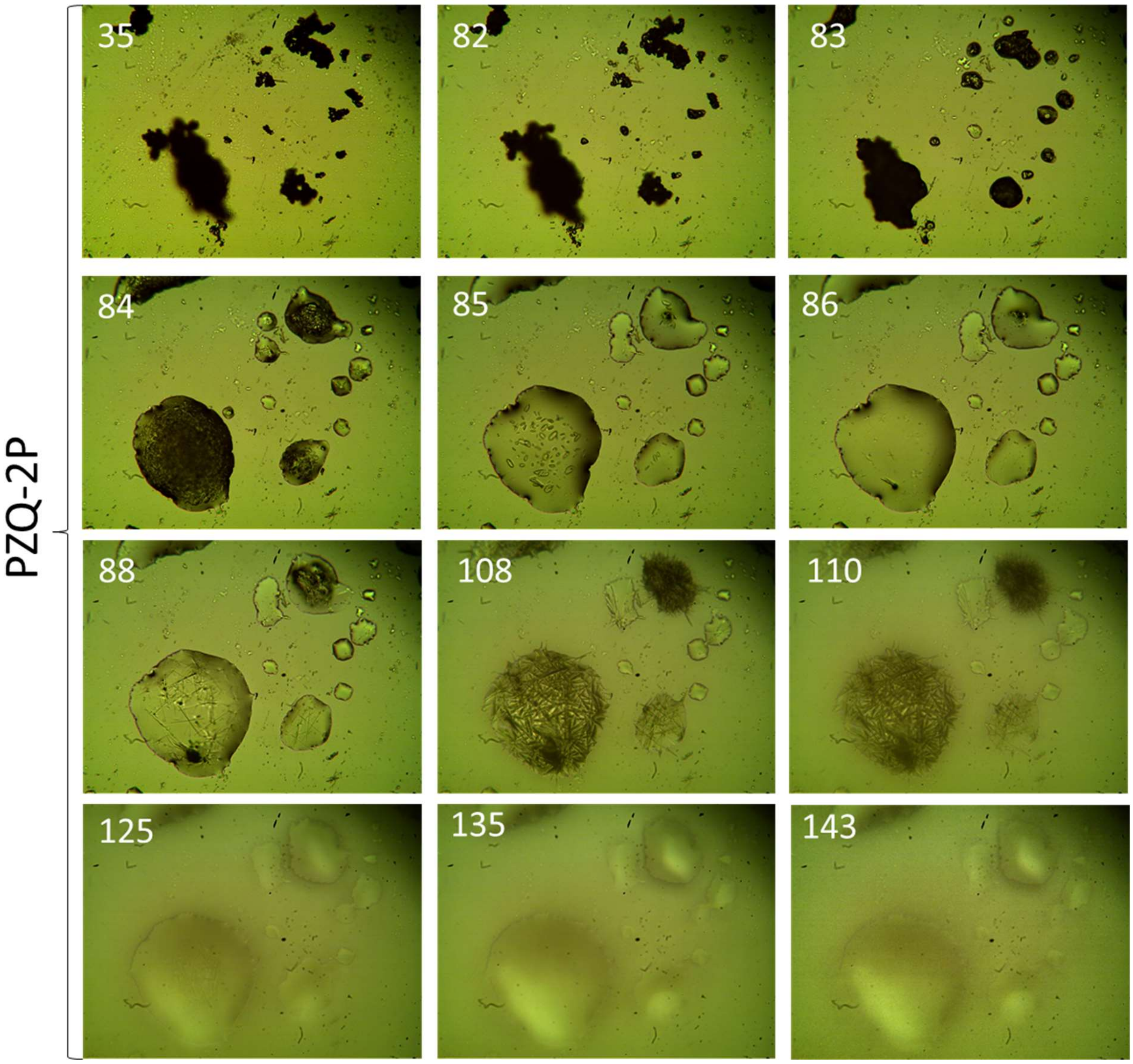


**Figure S4.** Optical microscopy images of PZQ-AA and PZQ-2P upon heating (with indication of operating temperature in each image). (Magnification 100x).

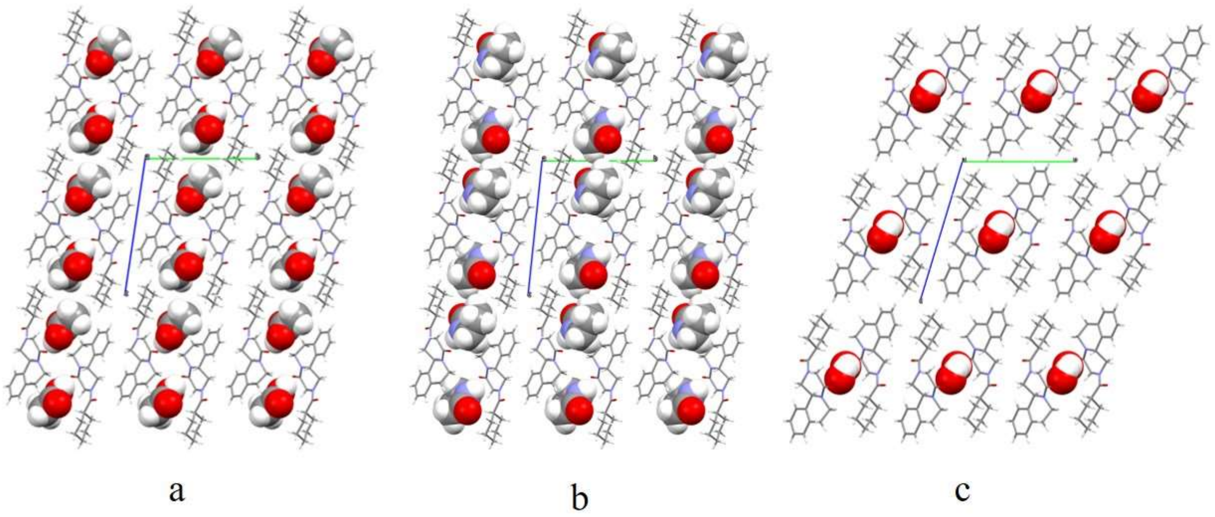


**Figure S5.** Crystal packing comparison along a axis of a) PZQ-AA , b) PZQ-2P and c) PZQ hemihydrate form.

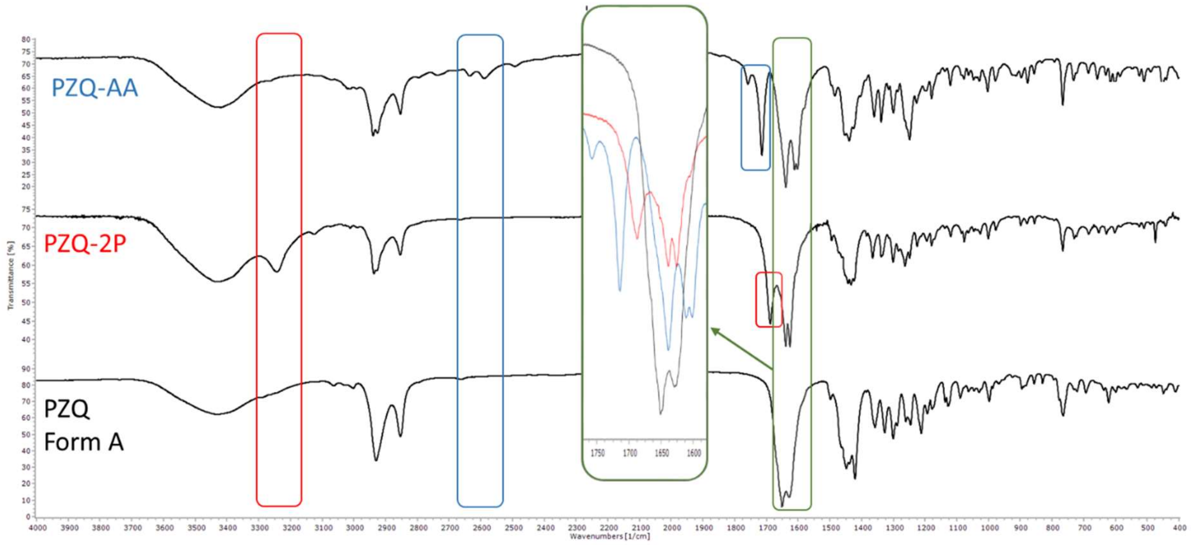


**Figure S6.** FT-IR spectra of PZQ-AA (blue), PZQ-2P (red) ad raw PZQ form A (green). Frames highlight with above-mentioned different colors diagnostic regions

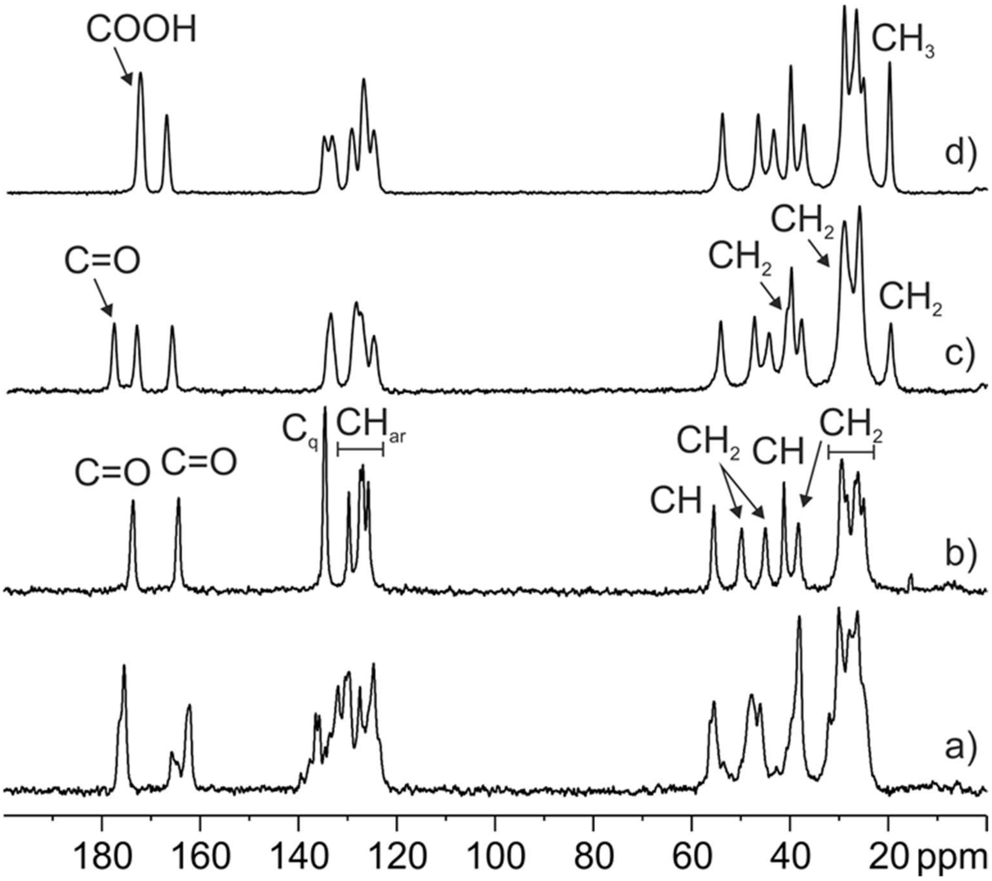


**Figure S7.** 13C (100.65 MHz) CPMAS SSNMR spectra of (a) PZQ form A, (b) PZQ form B, (c) PZQ-2P, and (d) PZQ-AA acquired at 12 kHz. Relevant assignments are reported for the PZQ molecule (b) and for the solvent molecules (c and d) resonances.

**Table S1.** Calculated shift / ppm and calculate shielding / ppm (for atom numbering please refer to Scheme).

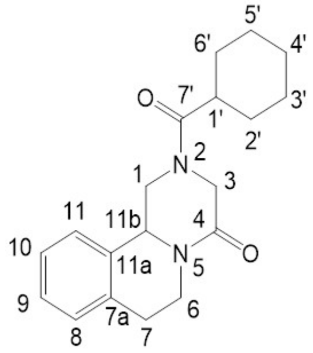


| | Acetic Acid solvate | | 2-Pyrrolidone solvate | |
|---|---|---|---|---|
| Atom | Calculate shielding / ppm | Calculated shift / ppm | Calculate shielding / ppm | Calculated shift / ppm |
| 7' | −1,68 | 172,68 | −2,2248 | 173,22 |
| 4 | 3,08 | 167,92 | 4,342 | 166,66 |
| 11a | 35,04 | 135,96 | 35,2514 | 135,75 |
| 7a | 32,97 | 138,03 | 33,8638 | 137,14 |
| 8 | 38,70 | 132,30 | 39,8349 | 131,17 |
| 11 | 44,17 | 126,84 | 44,1118 | 126,89 |
| 9 | 42,34 | 128,66 | 40,1189 | 130,88 |
| 10 | 42,09 | 128,91 | 40,9029 | 130,10 |
| 11b | 117,58 | 53,42 | 116,1831 | 54,82 |
| 3 | 125,49 | 45,51 | 124,7734 | 46,23 |
| 1 | 129,93 | 41,07 | 128,1975 | 42,80 |
| 1' | 131,94 | 39,06 | 131,3745 | 39,63 |
| 6 | 136,29 | 34,71 | 136,1598 | 34,84 |
| 6' | 143,32 | 27,68 | 143,7767 | 27,22 |
| 2' | 143,62 | 27,38 | 142,5896 | 28,41 |
| 7 | 145,70 | 25,30 | 144,7837 | 26,22 |
| 4' | 145,39 | 25,61 | 145,9471 | 25,05 |
| 3' | 145,54 | 25,46 | 146,6308 | 24,37 |
| 5' | 147,40 | 23,60 | 146,4202 | 24,58 |
| AA COOH | −6,13 | 177,13 | | |
| AA $CH_3$ | 152,97 | 18,03 | | |
| 2P $CH_2$ | | | 151,9089 | 19,09 |
| 2P $CH_2$ | | | 142,8449 | 28,16 |
| 2P $CH_2$ | | | 129,5215 | 41,48 |
| 2P CONH | | | −7,9436 | 178,94 |

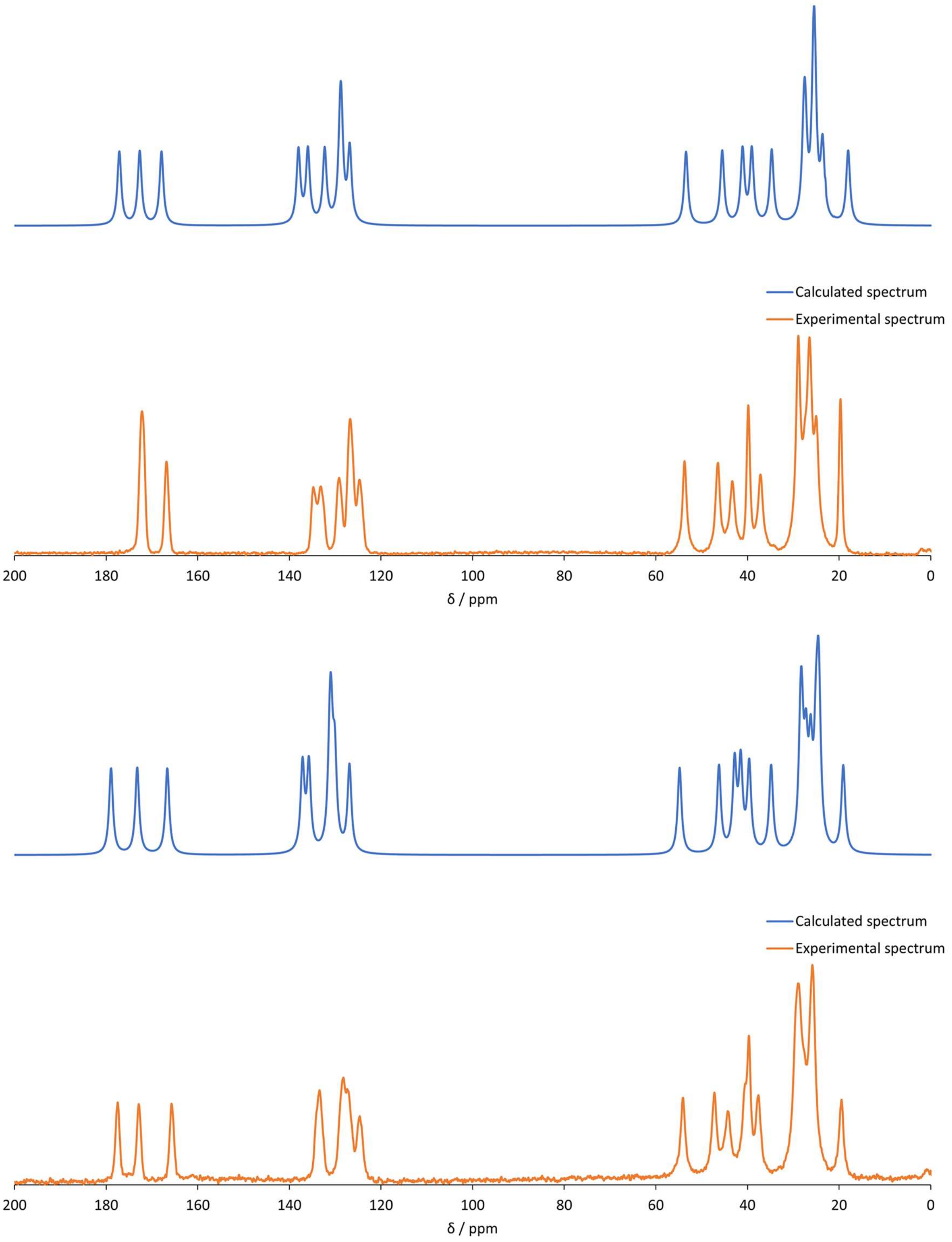


**Figure S8.** Comparison of experimental and calculated 13C SSNMR spectra of PZQ-AA (top) and PZQ-2P (bottom). The only significant disagreement is found for the COOH signal, at 177.1 and 172.1 ppm in the PZQ-AA calculated and experimental spectrum, respectively. The 5 ppm difference may be attributed to the difficulty in modelling the electronic environment around an acidic ionizable proton.

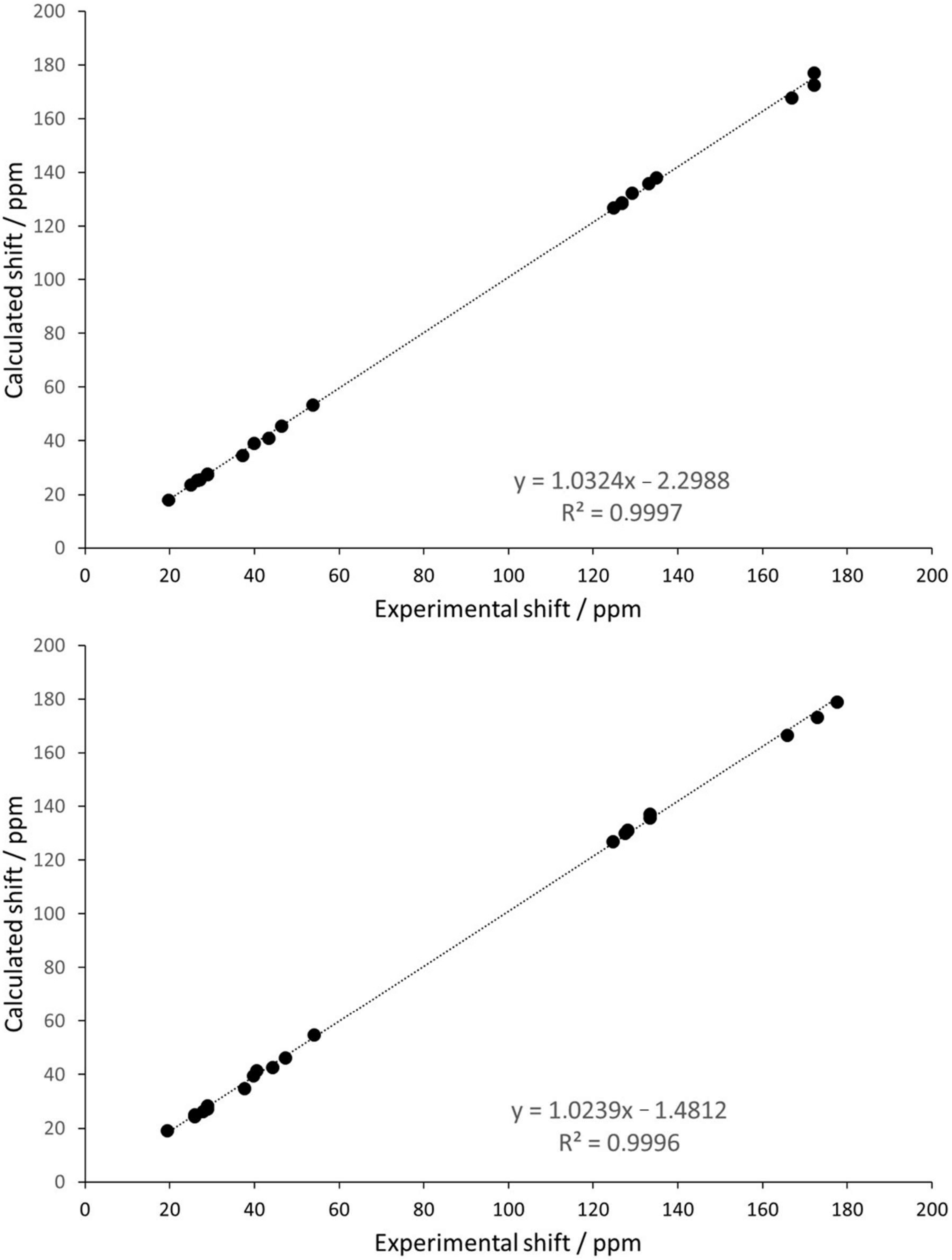


**Figure S9.** Quantitative comparison of experimental and calculated 13C SSNMR spectra of PZQ-AA (top) and PZQ-2P (bottom).
